# Blended psychological interventions for emotional disorders in youth: acceptability and intention to use in a sample of Portuguese psychologists

**DOI:** 10.47626/2237-6089-2023-0770

**Published:** 2025-09-09

**Authors:** Bárbara Gomes-Pereira, Ana C. Góis, Ana Maria Pereira, Brígida Caiado, Diana Santos, Helena Moreira, Ana Isabel Pereira

**Affiliations:** 1 Universidade de Coimbra Faculdade de Psicologia e de Ciências da Educação Centro de Investigação em Neuropsicologia e Intervenção Cognitivo-Comportamental Coimbra Portugal Centro de Investigação em Neuropsicologia e Intervenção Cognitivo-Comportamental, Faculdade de Psicologia e de Ciências da Educação, Universidade de Coimbra, Coimbra, Portugal.; 2 Universidade de Lisboa Faculdade de Psicologia Lisboa Portugal Faculdade de Psicologia, Universidade de Lisboa, Lisboa, Portugal.

**Keywords:** Acceptance, attitudes and expectancies, blended interventions, intention to use, psychologists, youth emotional disorders

## Abstract

**Objectives:**

The increase in mental health problems among youth highlights the need for accessible and cost-effective psychological interventions. Blended interventions, which combine face-to-face and online sessions, can be an adequate response to the increase in demand for youth mental health services. Although this can be a promising approach, effective dissemination depends on its acceptability to professionals. This study aimed to explore the acceptability of and intention to use blended interventions among psychologists working with children with emotional disorders and to examine predictors of these variables, including previous knowledge, expectancies (i.e., performance expectancy, effort expectancy, social influence, and facilitating conditions), and attitudes toward evidence-based practices (EBPs).

**Methods:**

The sample comprised 76 Portuguese psychologists (M_age_ = 37.26 years, standard deviation [SD] = 10.47; 92.1% female) working in youth mental health services. The participants completed an online questionnaire to evaluate the different dimensions included in the study.

**Results:**

The results showed that most participants demonstrated moderate to high acceptance of blended psychological interventions for emotional disorders in youth and intended to use them in the future. Regression analysis showed that performance expectancy and positive attitudes toward EBPs were significant predictors of acceptance of blended interventions and that social influence was a significant predictor of both acceptance of and intention to use blended interventions.

**Conclusion:**

These results emphasize the importance of sharing the findings of blended interventions, changing professionals’ attitudes toward EBPs, and of collaborating more closely with organizations and institutions to advance standards that encourage the adoption of this intervention format.

## Introduction

Mental health problems among children and adolescents represent a growing public health concern because of their long-lasting negative effects.^1^ Emotional disorders in particular (a term that groups together anxiety disorders, anxiety-related disorders, and depressive disorders),^2,3^ have a negative effect on children's development^4,5^ and quality of life^6^ and impact several different domains of childhood life, contributing to poor academic performance or social functioning.^7^ In fact, a meta-analysis conducted by Polanczyk et al.^8^ with data from 27 countries reported worldwide prevalence of 6.5% for any anxiety disorder and 2.6% for any depressive disorder in youth, and recent studies suggest that the coronavirus disease 2019 (COVID-19) pandemic has increased the prevalence of these problems in youth.^9^

### Online and blended psychological interventions for youth

Despite widespread recognition of the importance of mental health promotion and prevention of mental disorders in children and adolescents, there is still an enormous disparity between the available resources and actual access to mental healthcare.^10,11^ Among the obstacles to obtaining proper mental health care are low socioeconomic resources, stigma, and accessibility problems,^12^ such as geographic distance to mental health services, lack of time (of both patients and professionals), long waiting times, and service costs.^13,14^ One may argue that relying only on conventional intervention delivery methods is insufficient and online interventions may help overcome these obstacles.

Online psychological interventions do not require participants to travel to sessions. They may also feel that their privacy is protected with this type of intervention, allaying any concerns about the stigma associated with face-to-face therapy.^15^ Previous research in adult populations has shown that online cognitive behavioral therapy (CBT), especially with therapist guidance, is efficacious when used for the treatment of emotional disorders^16,17^ (e.g., depression). Some self-guided interventions for children with anxiety symptoms have also been developed, such as Lumi Nova (BfB labs)^18^ and BRAVE-online.^19^ Research has shown that these interventions potentially reduce symptoms,^20,21^ and have been accepted by children, parents and clinicians.^22^ However, several limitations emerge in online self-guided interventions, such as absence of human contact, unreliability and failure of technological equipment, limited internet access, or a need for greater participant autonomy, since there are no face-to-face sessions to clarify questions that may arise.^23^

A blended format that combines online sessions and face-to-face sessions can overcome some of the limitations of self-guided online interventions, enabling interventions that are better tailored to the child's needs and development of a relationship between patient and therapist through face-to-face sessions.^24^ The blended format also offers cost-effectiveness, since it maintains some of the characteristics of the online format, allowing for greater accessibility to treatment.^25^ Therefore, blended therapy is suggested as a promising innovation for the psychotherapeutic setting.^26^

### Predictors of acceptability of and intention to use blended psychological interventions for children with emotional disorders

One of the most critical factors in determining whether patients use blended programs is the therapists’ acceptance of this type of delivery format. Acceptability can be described as "the extent to which people delivering or receiving a healthcare intervention consider it to be appropriate, based on anticipated or experienced cognitive and emotional responses to the intervention."^27^ Some studies aiming to examine attitudes toward blended therapy have shown that blended treatment is generally accepted, although psychotherapists do not prefer web-based or blended therapy over face-to-face therapy.^28,29^ To the best of our knowledge, only one published study has addressed psychologists’ attitudes toward online psychological interventions for adults in Portugal. Mendes-Santos et al.^30^ found that most Portuguese psychologists had a slightly negative/neutral view toward such treatments and were unfamiliar with them, had no specific training, and had no prior experience utilizing online therapies. These results from Portugal starkly contrast with countries such as Australia,^31^ the United Kingdom, and Sweden,^32^ where use of internet interventions is widely disseminated. As conceptualized by Topooco et al.,^32^ Portugal may be included in the "learners" category in this domain, given the very limited current experience and practice of e-mental health in the country. In the abovementioned study,^30^ Mendes-Santos et al. also found that blended treatment interventions had greater acceptability than self-guided online interventions.

Attitudes toward manualized evidence-based treatment (EBT) for adults might also be an important determining factor of the acceptability of online and blended interventions for professionals,^33–35^ since they are usually structured and manualized. In general, there are still negative attitudes toward manualized EBTs, with several professionals perceiving them as less relevant to their clinical work than other factors^36^ (e.g., clinical experience) and not valuing, or only minimally valuing, the role of research in their clinical practice.^37^ According to earlier research, professionals’ negative attitudes toward web-based solutions were generally recognized as barriers to their effective uptake and recommendation.^33,38^ However, despite this evidence, there has been a growing movement toward acceptance of evidence-based practices (EBPs). For example, a study by Lilienfeld et al.^39^ showed that many or most mental health professionals had a reasonably positive view of EBPs and their usefulness in clinical practice. There is also evidence that therapists have an interest in and positive attitudes toward implementation of blended therapy^35^ in particular, which is considered a facilitator to uptake of these interventions.

Other factors, such as therapists’ knowledge of online and blended treatments and their prior usage^30^ have been proposed as potential predictors of acceptability and of whether therapists will use online therapy. Specifically, Mendes-Santos et al.^30^ identified lack of knowledge and training as one of the main obstacles to overcome in order to guarantee the successful implementation of online interventions.

The Unified Theory of Acceptance and Use of Technology (UTAUT)^40^ was developed to better understand the predictors of users’ intentions to use information technology (IT), such as an online psychological intervention, and their subsequent usage behavior. The UTAUT is based on the theory of reasoned action (TRA), the theory of planned behavior (TPB), and the social cognitive theory (SCT).^41^ According to this model, four cognitive dimensions may play a significant role as direct determinants of the intention to use an online intervention: performance expectancy (i.e., how much a person thinks the intervention will work and be beneficial); effort expectancy (i.e., the degree to which a person believes that adopting the intervention will be easy); social influence (i.e., how much a person believes that others believe they should use the intervention); and facilitating conditions (i.e., the degree to which an individual believes that an organizational and technical infrastructure exists to support the use of the system).

However, to the best of our knowledge, all the studies conducted on acceptability and intention to use of blended interventions among psychologists have only been carried out as a whole and not specifically for use with children. In other words, there is a need to understand whether professionals’ levels of acceptance and intention to use, as well as the predictors of these variables, change when the target population of the blended intervention is children with emotional disorders.

### The present study

In this study, we intended to explore the predictors of acceptability to Portuguese psychologists and of their intention to use blended interventions for children with emotional disorders. Specifically, we aimed to (1) describe psychologists’ prior knowledge and experience with blended interventions, as well as the level of acceptability and their intention to use these interventions, and (2) assess the role that psychologists’ expectations and attitudes toward manualized EBPs may have on their acceptance of and intention to use blended interventions for children with emotional problems.

## Method

### Participants

The study sample comprised 76 Portuguese psychologists (M_age_ = 37.26 years, SD = 10.47; 92.1% female). Most participants reported having a bachelor's or master's degree (88.2%), having a clinical and health psychology specialization (73.7%), and adopting a cognitive-behavioral approach (60.5%). Their detailed characteristics are presented in Table 1.

**Table 1:** Sociodemographic, academic, and professional background variables

		n (%)
Gender		
	Female	70 (92.1)
	Male	6 (7.9)
	
Professional work location		
	Urban	63 (82.9)
	Rural	13 (17.1)
	
Academic training		
	Bachelor's (5 years) or master's	67 (88.2)
	PhD	9 (11.8)
	
Specialization		
	Clinical and health psychology	56 (73.7)
	Educational psychology	17 (22.4)
	Organizational psychology	1 (1.3)
	Junior psychologists	5 (6.6)
	No specialization	7 (9.2)
	
Theoretical approach		
	Cognitive-behavioral	46 (60.5)
	Psychodynamic	10 (13.2)
	Systemic	7 (9.2)
	Integrative	9 (11.8)
	Humanist	3 (3.9)
	Other	1 (1.3)
	
Years of professional experience		
	0 -3	23 (30.3)
	4-15	26 (34.2)
	16-37	27 (35.5)
	
Practice Context		
	Central hospital	6 (7.9)
	Private hospital	1 (1.3)
	Primary care center	5 (6.6)
	Private practice	29 (38.2)
	School	19 (25.0)
	Other	16 (21.1)

### Ethical considerations

Ethical approval for the current study was obtained from the Ethics and Deontology Committee of the Faculdade de Psicologia of the Universidade de Lisboa and the Universidade de Coimbra (CEDI/23/06/2021).

### Procedures

The inclusion criteria were being a psychologist working in the field of child mental health (including psychologists working with adolescents or parents). All participating professionals completed the questionnaires via a data collection website (LimeSurvey^®^) between June 2021 and March 2022. The study and the survey link were shared on social media, on the website of the Portuguese Psychologists’ Association, and via e-mail. Before starting the study, participants were informed about the definition of blended interventions (i.e., those which combine online and face-to-face sessions) and informed about the main objectives of the study and assured that their responses would be anonymous. After reading the information about the study, participants had the option of going ahead and giving their consent to participate. A total of 220 psychologists started answering the online questionnaire, but only 76 completed it.

### Measures

#### Sociodemographic questionnaire

The first part of the questionnaire, which was developed based on previous sociodemographic questionnaires used by our research team, concentrated on gathering essential sociodemographic information. Participants were asked about age, sex, nationality, academic background, specialization in psychology, advanced specialization in psychology, main theoretical orientation of their interventions, professional activities performed, context and location of professional activities, district (region) of their workplace, and number of years of professional experience.

#### Previous experience with online and blended interventions

Psychologists were asked several questions regarding their previous experience with online and blended interventions, including questions such as "In your professional practice, have you ever implemented any online, or blended psychological intervention programs for children or adolescents?"; "In your professional practice, do you usually recommend or have you ever recommended the use/consultation of online resources to children, adolescents, or parents as a complement to the psychotherapeutic process?"; and "Which online resources do you usually recommend or have you ever recommended?"

#### Degree of knowledge of online and blended interventions

A short scale was developed for the current study to evaluate the psychologists’ level of knowledge about several aspects of online and blended interventions. The scale was composed of six items, rated on a Likert scale ranging from 1 (nonexistent) to 5 (high). The first three questions assessed knowledge about the content, mode of functioning, and existing research regarding self-guided online interventions (regardless of age group), and the remaining three questions asked the same questions regarding blended interventions. A principal component analysis found that a single factor emerged from the six items. Cronbach's alpha for the total score was 0.96.

#### Attitudes toward manualized EBP

The Portuguese version of the Evidence-Based Practice Attitude Scale (EBPAS)^42^ was used to assess psychologists’ attitudes toward manualized EBTs. The following explanation was provided in the instructions of the questionnaire: "Manualized therapy refers to any intervention that has specific guidelines and/or components that are outlined in a manual and/or that are to be followed in a structured/predetermined way." The questionnaire includes 15 items, which are rated on a five-point Likert scale (0 = strongly disagree to 4 = strongly agree), with higher scores indicating more favorable attitudes. Cronbach's alpha for the total scale was 0.73.

#### Expectancies towards blended psychological interventions for children with emotional disorders

A questionnaire, based on the UTAUT^40^ model, was specifically developed for the current study. Principal component analysis was performed to examine the factor structure of the questionnaire. Four factors emerged from the analysis: Performance Expectancy (seven items assessing the degree to which an individual believes that using the system will help him or her to attain gains in job performance/perceived usefulness, e.g., "A blended psychological intervention would increase the effectiveness of my clinical work"); Social Influence (four items assessing the degree to which an individual perceives that significant others believe he or she should use the system/subjective norms, e.g., "My superiors would support my decision to implement a blended psychological intervention"); Facilitating Conditions (seven items assessing the degree to which an individual believes that an organizational and technical infrastructure exists to support the use of the system/perceived behavioral control, e.g., "The institution where I work would provide the necessary means for me to implement the intervention"); and Effort Expectancy (two items assessing degree of ease associated with the use of the system, e.g., "Implementing a blended intervention would require too much of my time and energy"). The items are rated on a Likert scale ranging from 1 (strongly disagree) to 5 (strongly agree). Cronbach's alpha reliabilities were 0.82 for Performance Expectancy, 0.85 for Social Influence, 0.70 for Facilitating Conditions, and 0.65 for Effort Expectancy.

#### Perceived acceptability of blended psychological interventions for children with emotional disorders

A three-item questionnaire was developed for this study to assess the psychologists’ perceived usefulness of blended psychological interventions for children with mild and severe anxiety and/or mood problems (e.g., "To what extent do you think blended psychological interventions can help children aged 6 to 13 years with moderate anxiety or mood problems?"). Items were rated on a five-point Likert scale ranging from 1 (not at all) to 5 (very). Cronbach's alpha was found to be good (0.80).

#### Intention to use blended psychological interventions for children with emotional disorders

The intention of psychologists to use or recommend blended psychological interventions for children with emotional disorders was evaluated by two items: "If an empirically validated blended psychological intervention was available for children aged 6 to 13 years with anxiety or mood disorders, would you consider using it in your professional practice?" and "If an empirically validated blended psychological intervention was available for children aged 6 to 13 years with anxiety or mood disorders, would you consider recommending it to a colleague?" These two items were answered on a three-point Likert scale (0 = no; 1 = maybe; 2 = no) and have a Cronbach's alpha of 0.87.

### Data analyses

For the data analysis, we used the Statistical Package for the Social Sciences (SPSS) statistical analysis software, version 28, for Windows. Descriptive analyses were performed to characterize the sample in terms of sociodemographic dimensions and to evaluate professionals’ degree of knowledge, experience levels, and preferences with relation to online and blended psychological interventions. The reliability of the various questionnaires used was examined with Cronbach's alpha, for which values above 0.70 are recommended.^43^ Correlations between all the variables under study were also checked. Two stepwise regression analyses were performed to examine the predictors of 1) psychologists’ acceptability toward blended interventions and 2) psychologists’ intention to use these types of interventions. Years of professional experience were input into the first step, degree of knowledge was added to the second step, EBPAS was included in the third step, and the four UTAUT model elements were added in the final step of the model.

## Results

### Experience and knowledge about online or blended psychological interventions

The mean scores and SDs for knowledge regarding self-guided online and blended psychological interventions are presented in Table 2.

**Table 2:** Mean ratings for knowledge about online or blended psychological interventions

		Mean	SD
Self-guided online psychological interventions			
	Content	2.41	1.21
	How it works	2.49	1.24
	Research	2.20	1.20
		
Blended psychological interventions			
	Content	2.51	1.21
	How it works	2.64	1.28
	Research	2.39	1.28

SD = standard deviation.

Scale items are scored with Likert values between 1 (nonexistent) and 5 (high).

Most professionals did not know about online or blended psychological intervention programs for children or adolescents (93.4%). None of the 76 participants had ever implemented online or blended psychological intervention programs for children or adolescents.

In their professional practice, 34 (44.7%) out of the 76 participants had already recommended use/consultation of online resources (e.g., websites, forums, social networks) to children, adolescents, or parents to complement the therapeutic process. The resources most recommended were websites related to mental health or another specific theme (n=29, 38.2%). In addition, most professionals (88.2%) had used videoconferencing programs (e.g., Skype, Zoom, Teams) to conduct consultations.

#### Preliminary results

The majority of the professionals said they would consider using a blended intervention in their professional practice if it were available and would recommend it to a colleague (57.9 and 64.5%, respectively). Correlations between the predictors of the intention to use and acceptability of blended interventions and the levels of acceptance by professionals are presented in Table 3.

**Table 3:** Scale correlations and means

	1	2	3	4	5	6	7	8	9
1. Years of professional experience	-								
2. Degree of knowledge, total	-0.11	-							
3. EBPAS, total	-0.23*	0.08	-						
4. Performance expectancy	-0.16	0.10	0.57**	-					
5. Social influence	-0.04	0.28*	0.45**	0.45**	-				
6. Facilitating conditions	0.04	0.17	0.36**	0.40**	0.50**	-			
7. Effort expectancy	-0.04	0.09	-0.14	-0.06	-0.21	-0.05	-		
8. Acceptability	-0.20	0.16	0.65**	0.62**	0.58**	0.39**	-0.11	-	
9. Intention to use	0.01	0.21	0.38**	0.38**	0.49**	0.40**	-0.10	0.56**	-
Scale, mean (SD)	11.50 (9.98)	14.64 (6.75)	2.92 (0.46)	3.62 (0.68)	3.49 (0.68)	3.68 (0.67)	2.80 (0.76)	3.55 (0.70)	1.59 (0.50)

EBPAS = Evidence-Based Practice Attitude Scale; SD = standard deviation.

* p < 0.05;

** p < 0.001.

#### Predictors of acceptability and intention to use for blended psychological interventions for children with emotional disorders

Two stepwise regression analyses were performed to assess the prediction of variables related to the acceptability of and intention to use blended interventions. Regarding the perceived acceptability of blended interventions, the results showed significant effects of social influence and performance expectancy, meaning that higher levels of these dimensions were predictive of higher levels of acceptability. The results also revealed that attitude toward EBP was a significant predictor of the acceptability of blended interventions (Table 4).

**Table 4:** Summary of stepwise regression analysis for the acceptability of blended interventions

Variable	Step 1			Step 2			Step 3			Step 4		
	B	SE B	β	B	SE B	β	B	SE B	β	B	SE B	β
Years of professional experience	-0.01	0.01	-0.20	-0.01	0.01	-0.18	-0.00	0.01	-0.04	-0.00	0.01	-0.06
Degree of knowledge				0.02	0.01	0.14	0.01	0.01	0.11	0.00	0.01	0.02
EBPAS, total							0.98	0.14	0.64**	0.54	0.16	0.35*
Performance expectancy										0.29	0.10	0.28*
Social influence										0.29	0.11	0.28*
Facilitating conditions										0.02	0.10	0.02
Effort expectancy										0.01	0.08	0.01
F change		2.98			1.59			49.17***			5.74***	
R^2^ change		0.04			0.02			0.38			0.14	

EBPAS = Evidence-Based Practice Attitude Scale; SE B = standard error of the B coefficient.

** p < 0.01;

*** p < 0.001.

With regard to the intention to use blended interventions, the only significant predictor was social influence (Table 5).

**Table 5:** Summary of regression analysis for intention to use blended interventions

Variable	Step 1			Step 2			Step 3			Step 4		
	B	SE B	β	B	SE B	β	B	SE B	β	B	SE B	β
Years of professional experience	0.00	0.01	0.01	0.00	0.01	0.03	0.01	0.01	0.12	0.00	0.01	0.08
Degree of knowledge, total				0.02	0.01	0.22	0.02	0.01	0.20	0.01	0.01	0.10
EBPAS, total							0.43	0.12	0.39**	0.16	0.14	0.15
Performance expectancy										0.09	0.10	0.13
Social influence										0.20	0.10	0.26*
Facilitating conditions										0.11	0.09	0.15
Effort expectancy										-0.01	0.07	-0.02
F change		0.00			3.53			13.0***			3.02*	
R^2^ change		0.00			0.05			0.15			0.12	

EBPAS = Evidence-Based Practice Attitude Scale; SE B = standard error of the B coefficient.

* p < 0.05;

** p < 0.001.

## Discussion

The primary objective of this study was to investigate the level of acceptance and intention to use blended psychological interventions for treating emotional disorders in children among Portuguese psychologists and identify the effect of these psychologists’ expectations and attitudes toward manualized EBPs on these two variables.

The results showed that most psychologists were not familiar with online or blended interventions for children and adolescents with emotional disorders and none of them had experience implementing this type of intervention in their practice. These results align with previous studies (e.g., Mendes-Santos et al.^30^) and underline the need for more knowledge about and experience with the use of online interventions among Portuguese psychologists. As mentioned above, some authors have even indicated that Portugal may fall under the heading of a learner's group in this area, due to the country's lack of online mental health knowledge and usage.^32^ However, in the current study, most of the participants did have experience conducting online consultations. The fact that the sample was recruited during the COVID-19 pandemic may have influenced these results, since the large-scale self-quarantine and shelter-in-place orders led many nonemergency medical services to adopt telehealth solutions to continue serving their patients.^44,45^

In addition, it was possible to verify that the psychologists had moderate to high levels of acceptance of and intentions to use a blended intervention for children between 6 and 13 years old with emotional disorders. These results are in line with other studies carried out in recent years^46,47^ and with studies carried out specifically in Portugal, in which, as previously mentioned, mental health professionals showed a high acceptance rate in relation to the use of blended therapies compared to fully online therapies.^30^ It is important to note that none of these studies were carried out specifically on the use of these interventions for children and that, to our knowledge, this study is innovative in this age group. Although several studies have pointed out that there is still a long way to go, the truth is that the opinion of therapists regarding use of online interventions (mainly blended interventions) has been changing and is becoming increasingly positive. Part of this change can be justified by the impact that the COVID-19 pandemic has had around the world. This hypothesis is supported by authors such as Wind et al.,^48^ who considered the outbreak of the pandemic to be a "turning point" for e-mental health since it increased use of technologies for therapeutic purposes.

This study also explored potential predictors of psychologists’ acceptance of blended interventions. The results showed that years of experience and the level of knowledge of blended interventions did not predict psychologists’ acceptance and neither did facilitating conditions nor effort expectancy. Nevertheless, positive attitudes toward manualized EBPs, social influence (i.e., the degree to which a psychologist believes that significant others, such as work colleagues or superiors, consider that a blended strategy should be used to address children's emotional disorders), and performance expectancy (i.e., the degree to which a psychologist believes that using a blended intervention for children with an emotional disorder will be effective and useful in their clinical practice) were shown to be significant predictors of the acceptability of blended interventions. These results are in line with previous studies that point to performance expectancy and social influence as important predictors for increasing the acceptability of online interventions to practitioners.^40,49,50^ For instance, Philippi et al.^51^ reported that the main predictor of practitioners’ acceptance of using web-based and mobile interventions was indeed performance expectancy, and Venkatesh et al.^40^ suggested performance expectancy was the most critical predictor of eHealth acceptance. Regarding social influence, these results are in line with those found in pediatric health care, which showed that the positive social influence of peers and parents had a significant positive effect on eHealth experiences^49,50^ and this was identified as a facilitator of acceptance of eHealth interventions.^52^

The predictors of psychologists’ intention to use blended therapies were also explored in this study, and the only significant predictor was social influence. Considering other studies, this result does not agree with research conducted previously, which points to social influence as a nonsignificant variable of behavioral intention and points to performance expectancy as the most significant predictor.^53,54^ The apparent inconsistency in the results of these studies may be attributable, in part, to variations within the analyzed samples. Previous studies included both mental health counsellors and primary care psychologists, professional groups whose realities can significantly differ from those encompassed by the current study. Consequently, the impact derived from peer encouragement might not manifest in the same manner, potentially altering the value of social influence. This incongruity can be elucidated by the disparities in the samples, which encompass differences not only in professional background but also in cultural aspects, thereby contributing to the observed variability in the results. In addition, it is also worth considering that the limited knowledge and experience demonstrated by Portuguese professionals in utilizing online and blended therapies^30,44^ might render their intentions to use such interventions more reliant on social factors. The relatively low familiarity and expertise within the realm of blended therapies could lead to heightened dependency on social aspects when contemplating adoption of this intervention approach.

The fact that social influence has been shown to be a significant variable in both acceptance of and intention to use blended interventions suggests that it would be important to target this variable and to develop strategies that focus on it in order to increase it. The integration of specific training for blended interventions in professional or educational settings could be an effective way to increase psychologists’ acceptance of these interventions.

### Limitations

While this study contributes to the understanding of acceptance of and intention to use blended interventions in a Portuguese context, it also has some limitations that should be considered. The sample primarily consisted of cognitive-behavioral therapists (60.5%), thus caution is warranted when extrapolating the findings to all mental health professionals in Portugal, as certain areas may be underrepresented. Furthermore, the significant variation in years of professional experience, ranging from novice practitioners to those with no prior experience, may pose an additional limitation as it complicates direct comparisons. In future studies, greater equality between the various theoretical perspectives of the professionals under study is crucial because this is a factor that may greatly influence their preferences. A second limitation stems from significant participant attrition during the online questionnaire, likely due to its extensive nature. This dropout pattern, observed from the outset, aligns with prior research.^55^ Future studies may benefit from adopting strategies proposed in existing literature, such as a two-phase approach: the first gathering consent, contact, and demographic data, and the second focusing on survey completion.^55^ A third limitation relates to the fact that most of the measures used, although derived from the literature and demonstrating adequate reliability, were specifically adapted for this study. This adaptation may hinder comparison of the results with existing literature. Additionally, particular attention should be given to the two measures that contain few items (e.g., two or three items), as they may lead to less reliable conclusions. One additional limitation is derived from the fact that, in contrast to the longitudinal nature of the original UTAUT study,^40^ this study had a cross-sectional design. Given the scarcity of empirical research on UTAUT and technology adoption, it is imperative to conduct longitudinal studies to facilitate comparisons with Venkatesh's UTAUT^40^ investigations and enhance understanding of technology adoption and usage.

## Conclusions

With the increasing number of mental health problems in children and adolescents and the difficulty of accessing resources in the current healthcare system, it is essential to develop and disseminate new and cost-effective therapeutic solutions. A blended format combining online and face-to-face sessions can overcome some of these problems. This approach allows greater accessibility to treatment, is more adapted to children's needs, and encourages a closer therapeutic relationship. Therefore, evaluating the acceptance of and intention to use these interventions among mental health professionals, such as psychologists, as well as their predictors, is critical to better disseminating them among professionals and increasing the likelihood of usage.

Overall, this study showed that despite low knowledge and experience using online or blended interventions, psychologists’ acceptance of and attitudes toward these interventions are positive. In addition, the study showed that variables such as performance expectancy and social influence can predict higher levels of acceptance and intention to use. Based on these results and considering the positive levels of acceptance, it seems necessary to start training mental health professionals on the use of these interventions and allow them to gain knowledge and experience in using them.

## Data Availability

The data that support this study are available from the authors upon request.
